# Comparative transcriptomic analysis provides insight into carpel petaloidy in lotus (*Nelumbo nucifera*)

**DOI:** 10.7717/peerj.12322

**Published:** 2021-10-25

**Authors:** Zhongyuan Lin, Dingding Cao, Rebecca Njeri Damaris, Pingfang Yang

**Affiliations:** 1 Institute of Oceanography, Minjiang University, Fuzhou, China; 2 State Key Laboratory of Biocatalysis and Enzyme Engineering, School of Life Sciences, Hubei University, Wuhan, China

**Keywords:** *Nelumbo nucifera*, Flower morphology, Petaloidy, Floral organ, MADS-box

## Abstract

Lotus (*Nelumbo nucifera*) is a highly recognized flower with high ornamental value. Flower color and flower morphology are two main factors for flower lotus breeding. Petaloidy is a universal phenomenon in lotus flowers. However, the genetic regulation of floral organ petaloidy in lotus remains elusive. In this study, the transcriptomic analysis was performed among three organs, including petal, carpel petaloidy, and carpel in lotus. A total of 1,568 DEGs related to carpel petaloidy were identified. Our study identified one floral homeotic gene encoded by the MADS-box transcription factor, *AGAMOUS* (*AG*) as the candidate gene for petaloid in lotus. Meanwhile, a predicted labile boundary in floral organs of *N. nucifera* was hypothesized. In summary, our results explored the candidate genes related to carpel petaloidy, setting a theoretical basis for the molecular regulation of petaloid phenotype.

## Introduction

Lotus (*Nelumbo nucifera*) is an aquatic plant, which is widely cultivated as a food crop in East Asia. Additionally, it is also one of the famous traditional flowers, especially in China. Based on different breeding purposes, lotus is classified into three groups, namely seed lotus, rhizome lotus, and flower lotus (Guo, 2009). With its high ornamental value, the aim of breeding in flower lotus is performed under distinct flower colors and shapes (Wang & Zhang, 2005). Generally, a flower is constituted by four floral organs, including sepal, petal, stamen, and carpel in angiosperms (Zik & Irish, 2003). Thus, the different number of floral organs and diverse organ features form various flower morphologies. Meanwhile, few-petaled, double-petaled, duplicate-petaled, and all-double-petaled flowers were classified in lotus flower morphology (Kubo et al., 2009; Wang & Zhang, 2005). Seed lotus and rhizome lotus generally have normal floral patterns with few-petaled. The seed lotus flower has a normal pistil advantaged for seed harvesting while the rhizome lotus favors underground development with a few flowers. In particular, flowers with aberrant floral organs are preferred for ornamental purposes as they consist of all the classifications of flower morphology. In lotus, the peculiar flower patterns are mainly constructed by aberrant floral organs, such as petaloid stamen and petaloid carpel.

The petaloid phenomenon attracted research attention as early as 286 BC (Meyerowitz, Smyth & Bowman, 1989). Petaloid organs in locations other than the flower have been discovered and having petal-like morphology (Irish, 2009). Based on three floral homeotic mutations and genetic relationships between Arabidopsis and Antirrhinum, the typical ABC model has been widely accepted since the 1990s (Bowman, Smyth & Meyerowitz, 1991; Coen & Meyerowitz, 1991). Based on this model, petals are determined by A- and B-class genes; stamens are determined by B- and C-class genes, while carpels are determined by C-class genes. Most of the ABC model genes are MADS-box transcription factors, except one A-class gene encoded by *APETALA2* (*AP2*). Meanwhile, this model is also applicable to many monocot flowers after modifications, despite their differences in flower morphology (Dodsworth, 2017; Nakamura et al., 2005). B-function genes, including *APETALA3 /PISTILLATA* (*AP3/PI*), are essential in influencing petaloid (Dodsworth, 2017). The role of *AP3/PI* genes is specific in ‘petaloid’ traits under the sliding borders or fading borders models, which is different from the earlier models (Irish, 2009). B class genes are more broadly expressed across the floral meristem suggesting that the ‘fading borders’ model can explain the gradual transitions in floral organ phynotype (Soltis et al., 2007). During flower patterning, *AP3* and *AG* associate with *LEAFY* (*LFY*) after induction (Wu et al., 2012). The labile petal/stamen boundary was corresponded to play with sliding A/C boundary in rose (Dubois et al., 2010). Class C and A function genes have antagonism regulation (Huang et al., 2017). The war of different MADS-box genes for common partners to form complex is the regulatory mechanism in whorl boundary compartmentalization (Liu & Mara, 2010). Loss of C-class gene functions results in the substitution of petals for stamens and sepals for carpels (Yanofsky et al., 1990). The reduction in the expressions of *AGAMOUS* (*AG*), a C-function MADS-box gene homolog gene caused the double flower morphology in rose, *Thalictrum thalictroides*, and *Cyclamen persicum* (Dubois et al., 2010; Galimba et al., 2012; Tanaka et al., 2013). When the expression of *RABBIT EARS* (*RBE*) is down-regulated, the transcripts of *AG* are derepressed in floral and inflorescence meristems (Bao et al., 2004). The interactions between *WUSCHEL* (*WUS*) and *AG* are involved in floral determination while *AG* is a central gene in the genetic network of floral organ development (Lenhard et al., 2001). Other transcription factors might regulate floral organ formation by affecting the ABC model genes. However, the molecular mechanism of petaloidy is still not fully understood.

Previously, it was shown that the obscure expression of several candidate genes in boundaries of petal and stamen could result in stamen petaloidy formation (Lin et al., 2018), which might also be influenced by DNA methylation (Lin et al., 2019a). The transgenic lines of *AP3* promoter with hypermethylation possessed abnormal stamens and petals (Wang et al., 2019). DNA hypermethylation inhibits the expression of *RhAG* that controls petal numbers (Ma et al., 2015). DNA methylation affects plant development *via* gene regulation and silencing of the transposable element (Zhang, Lang & Zhu, 2018). Altered DNA methylation shows diverse phenotypes, like altered floral morphologies (Bräutigam & Cronk, 2018). Additionally, the latest studies on lotus were comprehensively reviewed, which revealed the absence of a detailed study on petaloid formation in *N. nucifera* (Lin et al., 2019b).

Lotus is a famous aquatic flowering plant, which has high ornamental value. The flowers of lotus have a variety of colors and multiple flower morphologies. With artificial selection, the double flower is popular for landscape architecture. Among them, ‘Sleeping beauty’ is a bowl lotus with a long flowering time and an abundant number of flowers, which also possess floral aberration. To obtain a comprehensive understanding of the petaloid formation in lotus, we used transcriptomic analyses among petal (P), carpel petaloidy (Cp), and carpel (C) from a bowl lotus ‘Sleeping Beauty’. The obtained results might provide some new insights into improving our understanding of petaloid carpel formation.

## Material and Methods

### Plant growth and sample collection

Lotus cultivar ‘Sleeping beauty’ was acquired from field genebank for lotus in Wuhan Botanical Garden, Chinese Academy of Sciences (WBGCAS), Hubei Province, China. The rhizomes of ‘Sleeping beauty’ were then separated into three plastic buckets (90 cm ×90 cm). Petal, stamen petaloidy, stamen, carpel, and carpel petaloidy were collected when the flowers bloomed on the fourth day of blossom. After sampling, the floral organs were snap-frozen in liquid nitrogen and stored at −80 °C until RNA extraction.

### Scanning electron microscope (SEM) observation

Petal and carpel petaloidy were collected from cultivar lotus ‘Sleeping Beauty’ on the fourth day of blossom. SEM was used to view epicuticular cells. In brief, the samples were dried at room temperature for one week. They were mounted on a stub using carbon tape. A vacuum was pulled and the samples were transferred to an ion sputter (MC1000). The samples were sputter coated for 90 s. with platinum and then transferred to specimen chamber of SEM (TM3030) in low vacuum condition.

### RNA isolation and sequencing

For each sample, more than 1.5 µg of total RNA was used. The RNA integrity number (RIN) of each sample was set above 6.5. Nine cDNA libraries were constructed, and Illumina sequenced by Beijing Novogene Bioinformatics Technology Co., Ltd. using the Illumina HiSeq 2500 high throughput sequencing platform. The transcriptome sequencing data was deposited in PRJNA524054.

### Data processing and bioinformatic analysis

After quality control with removing sequenced reads containing adapter, poly N, or low-quality sequences (Q <20), the remainder were termed as clean reads. Clean data was deposited in BMKCloud (https://www.biocloud.net/) for analysis. We mapped the data to the reference genome of *Nelumbo nucifera* (ASM1431973v1) (Li et al., 2021) using HiSat2 Software v2.0.4 (Kim, Langmead & Salzberg, 2015). Transcript assembly, differential expression, and divergent regulation were performed using Cufflinks v2.1.1 (Trapnell et al., 2010). The differentially expressed genes (DEGs) were carried out by using DESeq2 version 1.6.3 package (Love, Huber & Anders, 2014). The DEGs were identified by false discovery rate (FDR) ≤ 0.01 and a fold change ≥ 2. Any genes with an adjusted *P*-value <0.05 were assigned as differentially expressed. The Gene Ontology (GO) enrichment analysis of DEGs was carried out by the GOseq R packages 1.10.0. The statistical enrichment of DEGs in Kyoto Encyclopedia of Genes and Genomes (KEGG) pathways (FDR ≤ 0.01) was tested by KOBAS (KEGG Orthology-Based Annotation System) software. The heatmap was constructed by using Multiple Experiment Viewer software (MeV 4.9.0, https://sourceforge.net/projects/mev-tm4/files/mev-tm4/MeV%204.9.0/).

### Construction of cDNA and quantitative real-time PCR (qRT-PCR) gene expression analysis

Total RNAs were isolated using an RNA reagent (OminiPlant RNA Kit, CWBIO, China), and genomic DNA contamination was removed by treating with RNase-free DNaseI (Thermo, Shanghai, China). Primers used for qRT-PCR were listed in Table S1. The qRT-PCR reactions were performed using the SYBR Green Master Mix (BioRad, http://www.bio-rad.com/) as described by Lin et al. (2018). In brief, the qRT-PCR reactions were performed on the Bio-Rad CFX Connect. The reaction was initiated at 95 °C for 10 s, followed by 40 cycles of 95 °C for 15 s, 60 °C for 15 s and 72 °C for 30 s. Relative gene expressions were normalized by comparison with the expression of lotus *β*-actin (Nn1g06108), and analyzed using the 2^−ΔΔCT^ method. The data was indicated as mean ± SE.

### Statistical analysis

The data for transcriptome analysis and the qRT-RCR results were obtained from at least three independent biological experiments. The differential significance analysis was performed using analysis of variance (ANOVA) with Tukey’s multiple comparison tests in SPSS 22.0 (IBM Corp., Armonk, NY, USA). Figures were created using Sigma Plot 10.0 (Systat Software, Inc. Germany).

## Results

### Petaloid phenotype of *N. nucifera*

**Figure 1:**
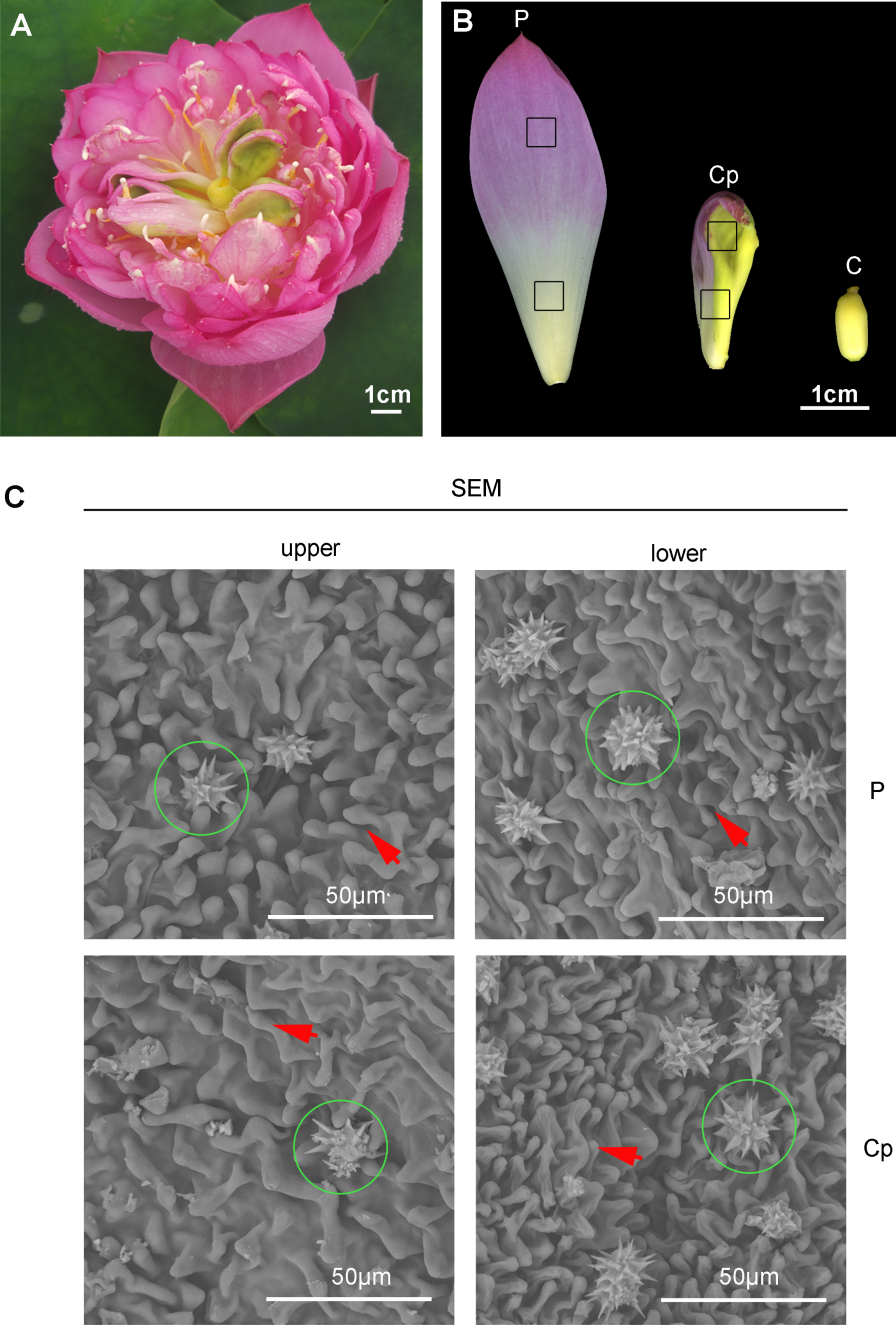
The flower of sacred lotus ‘Sleeping Beauty’. (A) The flower morphology of ‘Sleeping Beauty’ was imaged. (B) Three different floral organs, including petal (P), carpel petaloidy (Cp), and carpel (C) The samples of P, Cp, and C were collected on the fourth day of blossom. (C) Scanning electron microscope (SEM) observation of the epidermal structure of the petal-like organ. Mastoid cells and epidermal cells of upper and lower Cp and P were viewed, respectively. Mastoid cells are shown by arrow and wax crystals are marked in a green circle.

A special phenomenon in the flower shape of ‘Sleeping beauty’ occurs (Fig. 1A). Carpel petaloidy is one of the abnormal floral organs, which is defective for reproduction (Fig. 1A). The morphology of petal, carpel petaloidy, and carpel was shown in Fig. 1B. Scanning electron microscope observations of epidermal cells from petal and carpel petaloidy showed mastoid cells characteristic and wax crystal characteristic (Fig. 1C). The upper and lower epidermal cells with mastoid cells and wax crystal were shown to have similar shapes between P and Cp (Fig. 1C).

### Overview of the transcriptomic analysis

**Figure 2:**
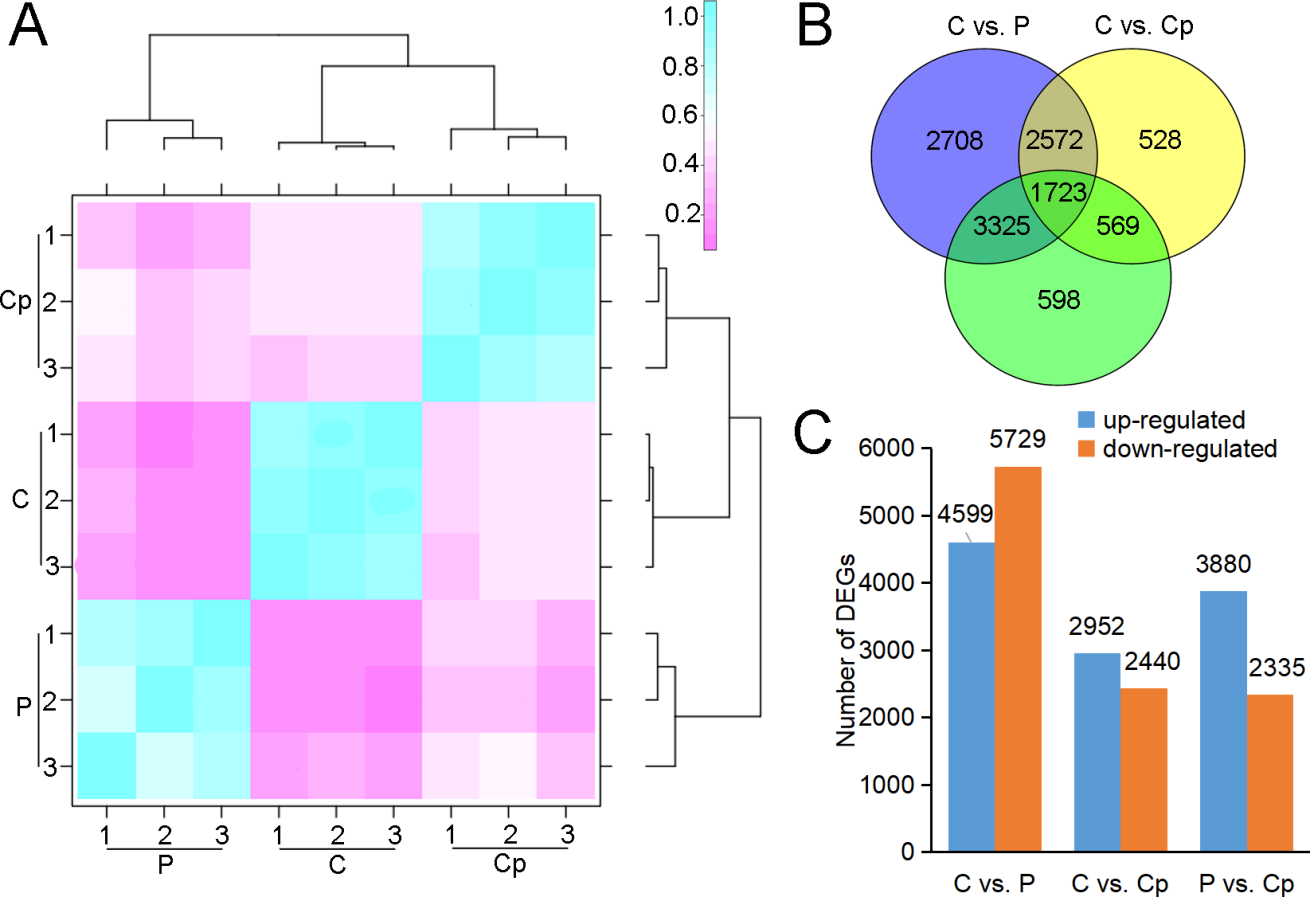
RNA-seq data and DEGs in P, Cp, and C. (A) Hierarchical clustering of nine samples based on correlation coefficient (*R*^2^) between each sample. The color panel represents the *R*^2^ values. (B) Venn diagram of the number of unique and common DEGs in the two comparisons (C *vs.* P, C *vs.* Cp, and P *vs.* Cp). (C) Statistics of up- and downregulated DEGs for each pairwise comparison. Up- and downregulated DEGs are displayed in blue and red, respectively.

RNA-seq was performed for three samples, including P, Cp, and C, with each one having three biological replicates. After sequencing quality control, a total of 59.86 Gb clean data was generated. The percentage of Q30 in each sample was no less than 92.09% (Table S2). 93.21–94.41% clean reads of each sample were mapped to the lotus genome (Li et al., 2021). Hierarchical clustering was performed for the samples based on the correlation coefficient (R^2^). P, Cp, and C were grouped in the respective clades, and their three biology replicates were clustered together (Fig. 2A). These implied that the samples and their duplicates were highly coherent. The total number of genes or transcripts from the samples was 48,572, out of which 1,896 were noted as new genes. All of them were annotated by COG class, GO, KEGG, KOG class, Pfam, Swiss Prot, eggNOG class and Nr (Table S3). After filtering out the genes with a low expression (FPKM < 5), DEGs were screened based on an absolute fold change of no less than two and an FDR ≤ 0.01. A total of 10,328 (C *vs.* P), 5,392 (C *vs.* Cp), and 6,215 (P *vs.* Cp) DEGs were detected, including 4,599, 2,952, and 3,880 up-regulated DEGs and 5,729, 2,440, and 2,335 down-regulated DEGs, respectively (Figs. 2B and 2C). Moreover, the Pearson relationships were performed with pair-wise comparison among these three tissues with their DEGs (Fig. S1). We found that the relationship of C and Cp, P and Cp had 0.812 and 0.8025, respectively (Fig. S1). The result suggested that carpel petaloidy was a mostly similar expression with petal and carpel.

### Identification of DEGs

**Figure 3:**
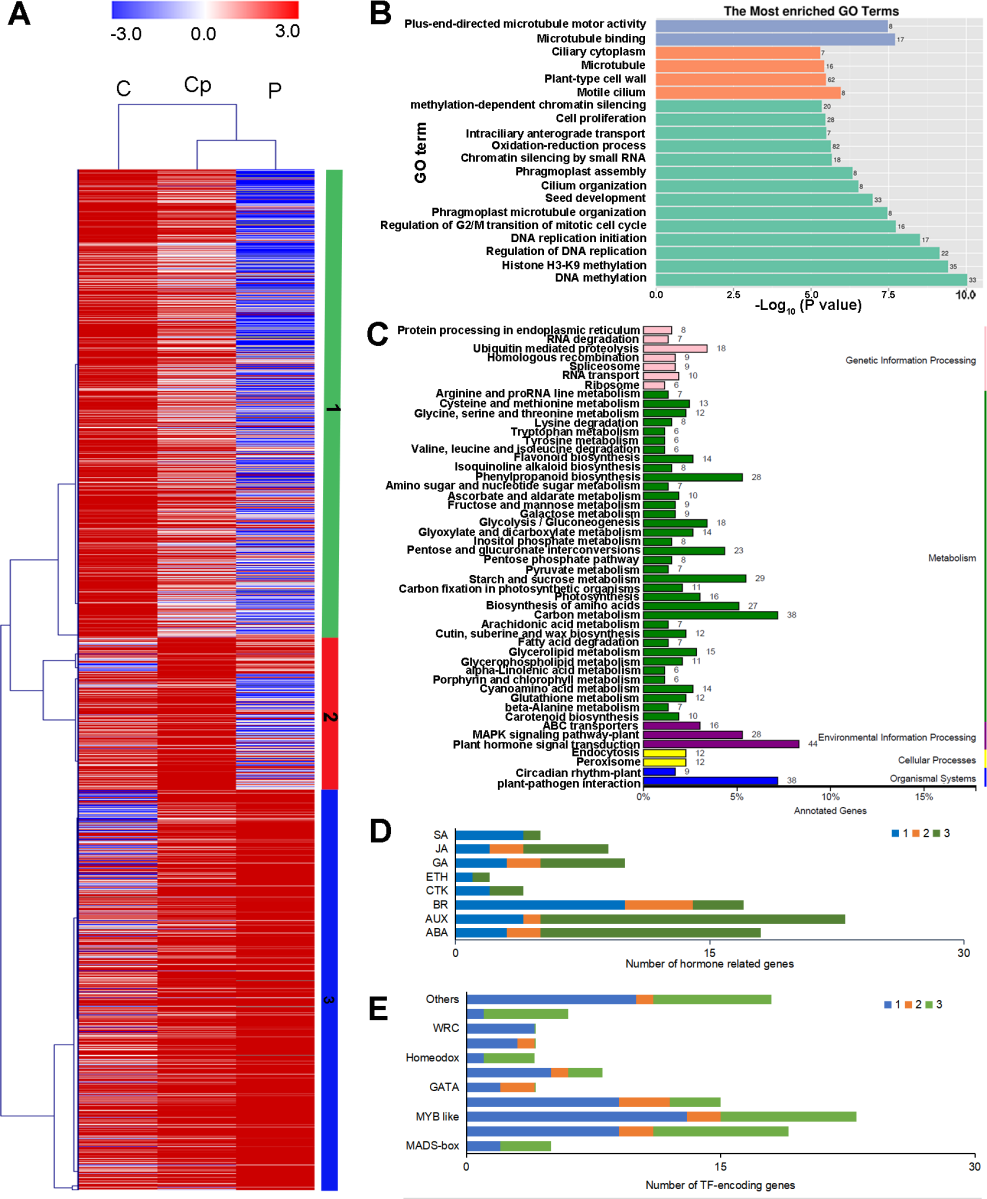
Hierarchical clustering analysis (A), enriched Go terms (B), and KEGG classification (C) of 1568 annotated DEGs among P, Cp, and C. (D) Distribution of TF families among cluster 1, cluster 2, and cluster 3. Cluster 1, 2, 3 were marked by green, red, and blue, respectively.

To investigate the regulation of gene expression in the carpel petaloidy, we subjected the expression values to pairwise comparisons, C *vs.* P, C *vs.* Cp, and P *vs.* Cp. In this study, a total of 12,023 DEGs were found, while only 14.33% of the DEGs (containing 1723 common genes) were common to these floral organs being selected (Fig. 2B). Furthermore, the number of DEGs that could be annotated was only 1,568 from the common DEGs in the different flower organs (Table S4). The hierarchical clustering analysis of their gene expression presented differentiated profiles in the carpel petaloidy group, with three large gene clusters (Fig. 3). The largest cluster (cluster 1 highlighted in green) consisted of 719 genes; while cluster 2 (highlighted in blue) contained 211 genes. Their expressions in Cp were between C and P; 638 genes (highlighted in red) were grouped in cluster 3 with most of them expressed more strongly in Cp (Fig. 3A). Annotation, GO-based, and KEGG-based functional analyses were performed (Figs. 3B and 3C). For all the chosen DEGs, DNA methylation and histone H3-K9 methylation were the top two most significantly enriched GO terms (Fig. 3B). Otherwise, plant hormone signal transduction, carbon metabolism, plant-pathogen interaction, starch and sucrose metabolism, phenylpropanoid biosynthesis, and MAPK signaling were the six top classifications (Fig. 3C). The most KEGG classifications were involved in metabolism (Fig. 3C). These indicated that metabolism might be significant in petaloidy formation. GO assignments was carried out to classify the functions of 1,568 DEGs, being categorized into three major groups (biological process, cellular component, and molecular function, Fig. S2A). Meanwhile, KEGG analysis was performed to explore the functional classification and pathway assignment of DEGs (Fig. S2B). Regulation of DNA replication, chloroplast thylakoid membrane, and cellular response to light stimulus were the most enriched GO term in cluster 1, cluster 2, and cluster 3, respectively (Fig. S3). Meanwhile, annotated genes by KEGG classification included plant hormone signal transduction, plant-pathogen interaction, biosynthesis of amino acids, and ubiquitin mediated proteolysis in cluster 1; Cluster 2 was contained photosynthesis, carbon metabolism and plant-pathogen interaction; For cluster 3, there were plant hormone signal transduction, phenylpropanoid biosynthesis, and glycerolipid metabolism, and plant-pathogen interaction (Fig. S4).

Furthermore, we also inspected the DEGs in more detail to elucidate the genetic regulation of the carpel petaloidy formation. After filtered with believing to be involved in carpel petaloidy, 1,568 DEGs were chosen for further analysis, could be the specific genes. Multiples of them were assigned as the auxin (AUX), abscisic acid (ABA), jasmonic acid (JA), gibberellin (GA), brassinosteroid (BR), ethylene (ETH), cytokinine (CTK), and salicylic acid (SA) pathways. In addition, numerous transcription factors (TFs) were also identified with these DEGs. Further, we identified the family-specific expression tendency of these hormone-related genes and TFs from three clusters (Figs. 3D and 3E). SA, CTK, and ETH were exclusively in cluster 1 and cluster 3, which included all mentioned phytohormone pathways. In cluster 2, it just had BR, AUX, GA, and ABA. Among these TFs, we found that MYB, zinc finger and bHLH like genes were the three most abundant represented by 23, 19, and 15 DEGs, respectively. Then followed by AP2 domain, NAC, and MADS were represented by eight, six, and five, respectively. The number of TFs assigned as GATA, homeobox, GRAS, and WRC genes was four, while less than two were grouped as other TFs. Cluster 1 was classified into all TF families with exclusively contained WRC. Most MYB-like was grouped in cluster 1. For cluster 3, it had three out of five MADS-box. These observations suggested that these hormone-related genes and TFs may function in a fixed mode.

### Hormone genes and TFs involved in carpel petaloidy

**Figure 4:**
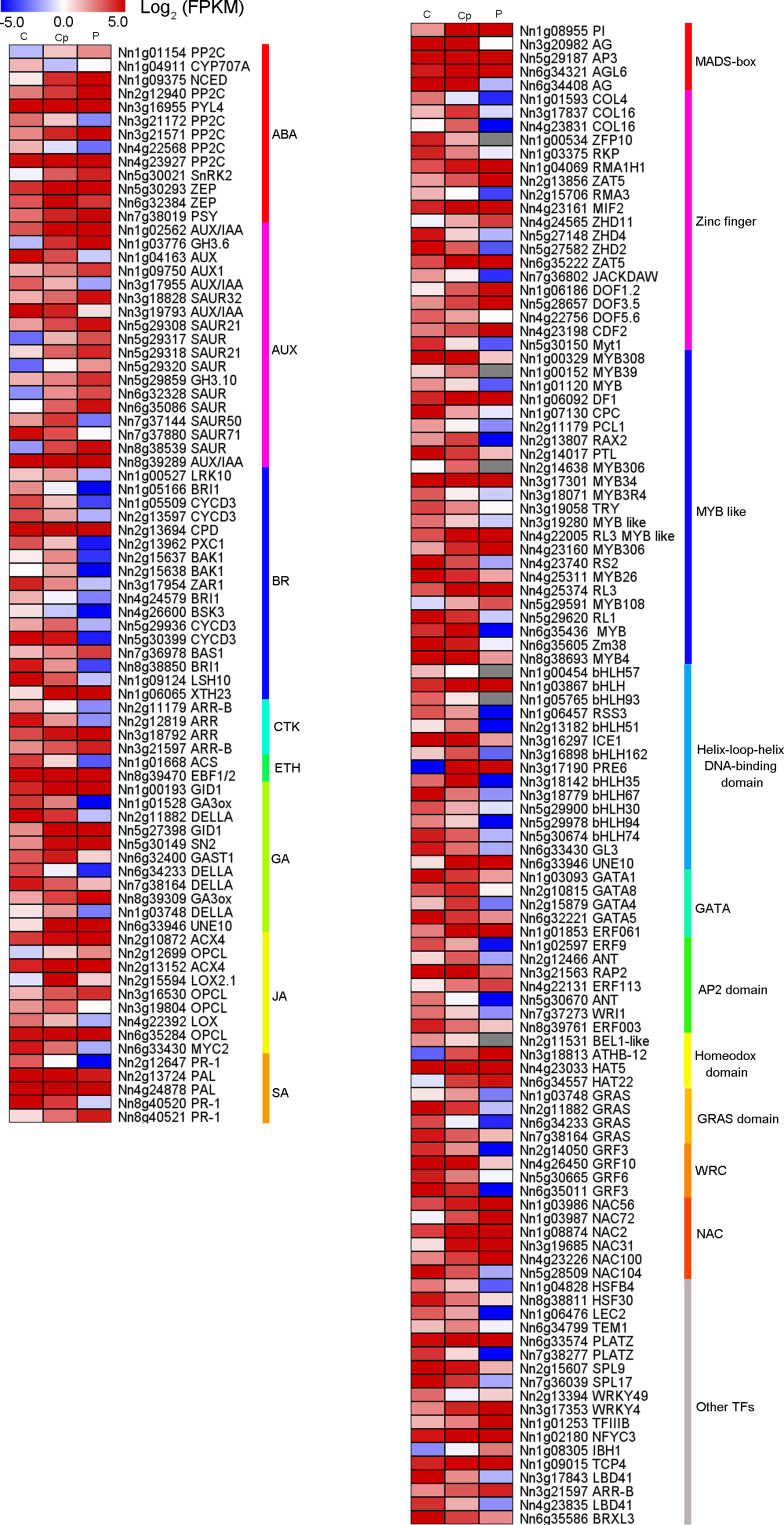
Heat maps of phytohormone-related genes and transcription factors (TFs) in comparison of three floral organs. (A) Seventy-nine phytohormone-related genes. (B) One hundred and ten transcription factors.

From the 1,568 DEGs, 79 DEGs were involved in phytohormone regulation (Fig. 4A). Those related to the AUX regulation pathways were the most enriched, suggesting that AUX pathway may play a significant role in carpel petaloidy. Notably, only 44 were assigned as plant hormone signal transduction by KEGG pathway annotation (Fig. 3C and Table S5). Fifteen of them were shown to be involved in AUX pathway, namely, four *AUX/IAA* (Nn1g02562, Nn3g17955, Nn5g29317, Nn3g19793, and Nn8g39289), nine *SAUR* (Nn3g18828, Nn5g29308, Nn5g29317, Nn5g29318, Nn5g29320, Nn6g32328, Nn6g35086, Nn7g37144, and Nn7g37880) and two *GH3* genes (Nn1g03776 and Nn8g39289). They were considerably varied in expression. The expression patterns of twelve out of fifteen genes were similar and peaked in petal, while Nn3g17955, Nn3g19793, and Nn7g37880 had an opposite pattern. Interestingly, a gene encoding auxin-responsive protein *SAUR* (Nn7g37144) was highly expressed in carpel petaloidy. These results showed that the AUX signal pathway might be significant to control carpel petaloidy.

In our data, there were 110 TFs out of the 1,568 DEGs, which most represented the MADS-box, Zinc finger, MYB, bHLH, AP2 domain and NAC family had a median expression in Cp compared with two other flower organs (Fig. 4B). Intriguingly, five DEGs encoding the MADS-box domain were found. Four of them belonged to the classical ABC model. They were three class B genes included Nn1g08955 (*PI*), NNU_08090 (*AP3*), and NNU_23351 (*AP3*). Additionally, Nn3g20982, and Nn6g34408 being *AG* homolog genes, member of Class C members had interesting expression patterns revealed by the FPKM value *via* transcriptome profiling in different floral organs (Fig. S5). Nn3g20982 and Nn6g34408 encoding for floral homeotic *AG* gene were found to have the lowest expression in petal compared with other flower tissues. In contrast, A-class genes (Nn4g23511, Nn1g04782, and Nn5g30360) and B-class genes (Nn1g08955, Nn5g29187, and Nn6g32205) expressed higher in petal. Additionally, based on the expression profiles, A-class genes, *AP1-like* (Nn4g23511) and *AP2-like* (Nn1g04782) had a similar expression, while C-class genes (Nn3g20982 and Nn6g34408) showed the opposite expression. These observations indicate that transcriptional regulatory mechanisms being implicated in carpel petaloidy is complex.

### The gene relative expression analysis

**Figure 5:**
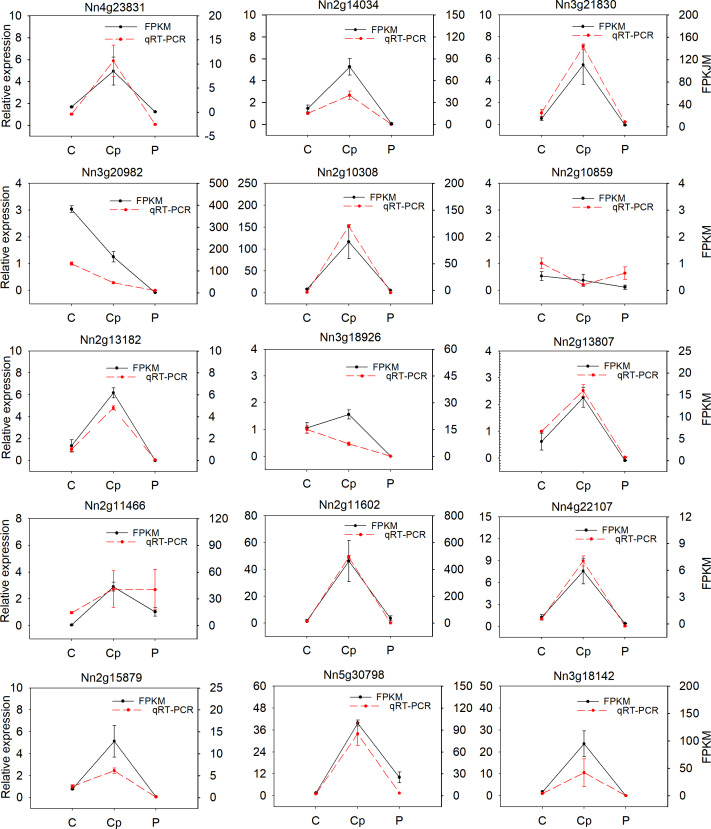
Relative expression of 15 differentially expressied genes (DEGs) and their FPKM value by RNA-seq. Values represent the means and standard errors (SEs) of three biological replicates. The expression level of each gene in one of three carpels was arbitrarily set as 1.0. FPKM is represented by black and qRT-PCR was show by red.

To verify the reliability of RNA-seq data, fifteen DEGs were randomly selected and subjected to qRT-PCR analysis in three floral organs (including P, Cp, and C). Most of the selected genes were consistent with RNA-seq data, except for the Nn2g10859, Nn3g18926, and Nn2g11466 (Fig. 5). The correlation coefficient (*R*^2^ = 0.825) between the results of RNA-seq and qRT-PCR was high (Fig. S6). These results proved the reliability of the transcriptome data.

## Discussion

The various phenotypes of lotus flowers have fundamental own critical ornamental value. The morphology of a flower is influenced by the aberrant floral organs, especially petaloid. Cultivar lotus ‘Sleeping Beauty’ has various abnormal floral organs similar to petals from the homeotic transformation of stamen and carpel. A previous study reported the existence of the petaloid stamen and petaloid sepal (Tian et al., 2016). However, a few studies are focusing on carpel or pistil petaloid formation. The carpel petaloidy is a unique phenomenon in lotus. To explore the mechanism of carpel petaloid formation, comparative transcriptomic analysis was performed in P, Cp, and C. This will enable the expansion of our understanding of flower development and petaloid carpel formation in lotus.

Currently, the lotus genome of ‘China Antique’ had been sequenced and released providing a research basis for omic study and breeding (Ming et al., 2013; Li et al., 2021). DEGs involved in petaloid carpel were identified with transcriptomic analysis in P, Cp, and C. For the above candidate genes (Fig. 4), they are almost certainly involved in the regulatory network of flower development, but their connections with floral organ formation have not been verified.

In model plants, a large number of transcription factors were reported to be involved in floral development, such as MADS-box, MYB, and bHLH (Dubos et al., 2010; Heijmans, Morel & Vandenbussche, 2012b; Tiancong et al., 2015). MADS-box transcription factors play key roles in controlling the morphogenesis of floral organs. *AGAMOUS* (*AG*) is involved in the regulation of stamen and carpel formation and development in *Arabidopsis* (Drews, Bowman & Meyerowitz, 1991; Yanofsky et al., 1990). Owing to A-class genes being expanded to the inner whorl, the transformation of stamens to petals is similar to a result of mutation of *AG*. In contrast, ectopic expression of *AG* in the outer whorl causes sepal carpeliod and petal stamenoid (Tzeng, Chen & Yang, 2002). *AG* homeotic gene has been universally identified in many plants, such as rose, petunia, *Thalictrum thalictroides*, *Prunus lannesiana* and *Medicago truncatula* (Dubois et al., 2010; Galimba et al., 2012; Heijmans et al., 2012a; Liu et al., 2013; Zhu et al., 2018). These show that the homologous *AG* pattern of expressions and regulation of stamen and carpel identity are conserved. In our study, notably, the candidate genes (Nn3g20982 and Nn6g34408) are *AG* homolog, belonging to MADS-box family. *AGs* were among the DEGs with lower expression in stamen petaloidy than in stamen. Meanwhile, their expression in carpel petaloidy was less than that in carpel and lowest in petal. These results show that a declined expression of *AG* in the inner whorl results in carpel petaloidy formation and breaks down the gene expression boundary. In the previous study, we performed transcriptome analysis for stamen petaloidy showing that several MADS-box genes, including *AG,* were found to be possibly involved in floral organ specification (Lin et al., 2018). Epigenetic regulation may influence carpel petaloid formation (Fig. 3B). DNA methylation and histone methylation may transmit genetic information through synergic action. The DNA methylation level of *RhAG* regulated its expression to determine the petal number (Ma et al., 2015). DNA methylation status was different in three floral organs of lotus (Lin et al., 2019a). In this study, we found that DNA methylation was one of the most enriched GO terms (Fig. 3B). Our finding suggested that DNA methylation might be an important factor involved in carpel petaloidy. The dynamics of DNA methylation-controlled floral organ petaloidy and their activated pathways will be carried out in future studies.

Plant hormones play a crucial role in the development process and organ genesis (Lee et al., 2019; Marsch-Martinez & De Folter, 2016). Multiple genes involved in much phytohormone metabolism or signaling pathways had distinctive expressions in the three samples. In this work, KEGG analysis of the DEGs unveiled enriched pathways for ‘plant hormone signal transduction’, which included 44 DEGs and fifteen of them related to AUX signaling pathway (Fig. 3C). Therefore, we specifically focused on the AUX pathway that regulates carpel petaloid formation. AUX has been reported to be important for gynoecium development (Marsch-Martinez & De Folter, 2016). Previous studies showed that the AUX is crucial for flower organ patterning (Huang & Irish, 2016; Robert et al., 2015). We found the AUX signaling pathway genes involved in the formation of petaloid stamen (Lin et al., 2018). Herein, we found that several genes involved in the AUX signal pathway. Most of them showed medium expression in Cp compared with C and P, except for one gene auxin-responsive protein *SAUR* (Nn7g37144) that had peaked transcript in Cp (Fig. 4A). Besides, two different expression patterns among these AUX signal pathway genes suggested the existence of a complicated regulation network among them that might control carpel petaloidy phenomena. Auxs are involved in flower organs identity through their interactions with other phyothormones, such as ABA, BR, ETH, GA, JA, and SA. Those, together contribute to flower development (Cheng & Zhao, 2007; Chandler, 2011). The ABA, AUX, GA, and JA signaling pathway DEGs were most up-regulated in Cp compared with C. However, the DEGs involved in SA signaling ptathys were largely down-regulated. The different hormone pathways play important roles in different aspects of floral organ formation. Almost all hormone pathways were found to modulate by TFs (Pajoro et al., 2014).

**Figure 6:**
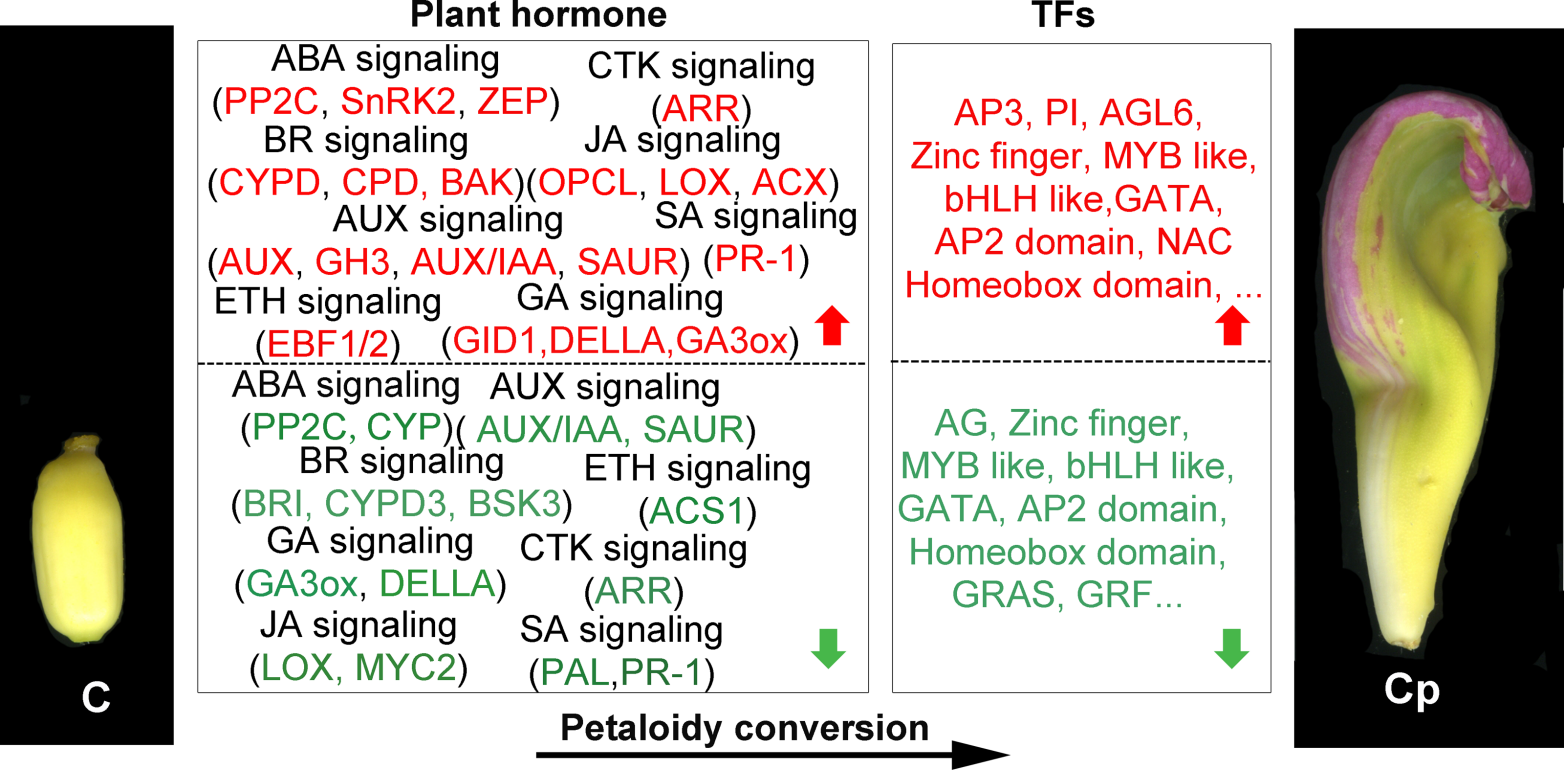
Summary of transcription-level regulation of carpel petaloidy. Differentially expressed genes (DEGs) may regulate carpel petaloidy (those with more than a one-fold change in expression, FDR < 0.05). DEGs were either up-regulated (red) or down-regulated (green) as petaloid formation.

Dozens of TFs were found that they showed distinct enrichments in different floral organs of louts. 110 TFs out of 1568 DEGs were chosen for further analysis. They represent multiple roles in different floral organs. For some GA-regulated network, involving homeotic genes are linked to the shaping of floral organs (Chandler, 2011). AG induced gibberellin biosynthesis enzyme GA3ox (Gómez-Mena et al., 2005). These consistent with AG and GA3ox having the same trend, which is dwon-regulation in carpel shifts to petaloidy carpel. Carpel development related genes are often repressed by AP3/PI and promoted by AG (ÓMaoiléidigh et al., 2013). It has been demonstrated that GATA family genes participated in carpel development, and repressed by AP3/PI in petal (Mara & Irish, 2008; Zhang et al., 2013). JA biosynthesis gene activated by GA regulates MYB like TFs through DELLA repression (Cheng et al., 2009). AP2 domain TFs were demonstrated to regulate the development of flower and seed (Okamuro et al., 1997). In lotus, we have reported the 121 AP/ERF proteins, with *NnADAP* effect the lotus rhizome development (Cao et al., 2021). However, the mechanisms to regulate the shape of floral organs are still unclear. Here, among AP2-like TFs, they showed that the distinct regulation styles were applied in C and Cp. BEL1-like homeodomain protein associate with a co-repressor complex to down-regulate AG during flower development. In our data, BEL1 like (Nn2g11531) has a similar expression with AG (Nn3g20982). Loss of several homeobox genes could alter floral patterning (Yu, Patibanda & Smith, 2009). Deficient two related BEL1-like homeobox genes (*PNY* and *PNF*) can cause misexpression of another homeobox gene *(ATH1*), whose is down-regulated GA pathway signaling and increased JA biosynthetic genes, and then activation of *LFY*, *AP1*, and *CAL* (Khan et al., 2015). These suggest that homeobox genes are essential for flower organs developement. These DEGs feasible regulated carpel to carpel petaloidy transformation is outlined in Fig. 6.

Few-petaled, double-petaled, duplicate-petaled, and all-double-petaled groups were systematized for the flower morphologies in lotus (Wang & Zhang, 2005). The different number of petals in cultivar lotus was generated by breeder domestication. For traditional breeding and production, they only focus on their aims of improving plants’ potential values. Factors in the molecular mechanisms on how they are modulated are still unclear. In normal floral morphology, dicotyledonous plants’ floral organs are located in four whorls in an orderly manner. Boundaries exhibit between floral organs within whorls (Lampugnani, Kilinc & Smyth, 2012). The labile boundary was previously found in rose flower and that the expression pattern of *AG* is responsible for morphological diversity (Dubois et al., 2010).

**Figure 7:**
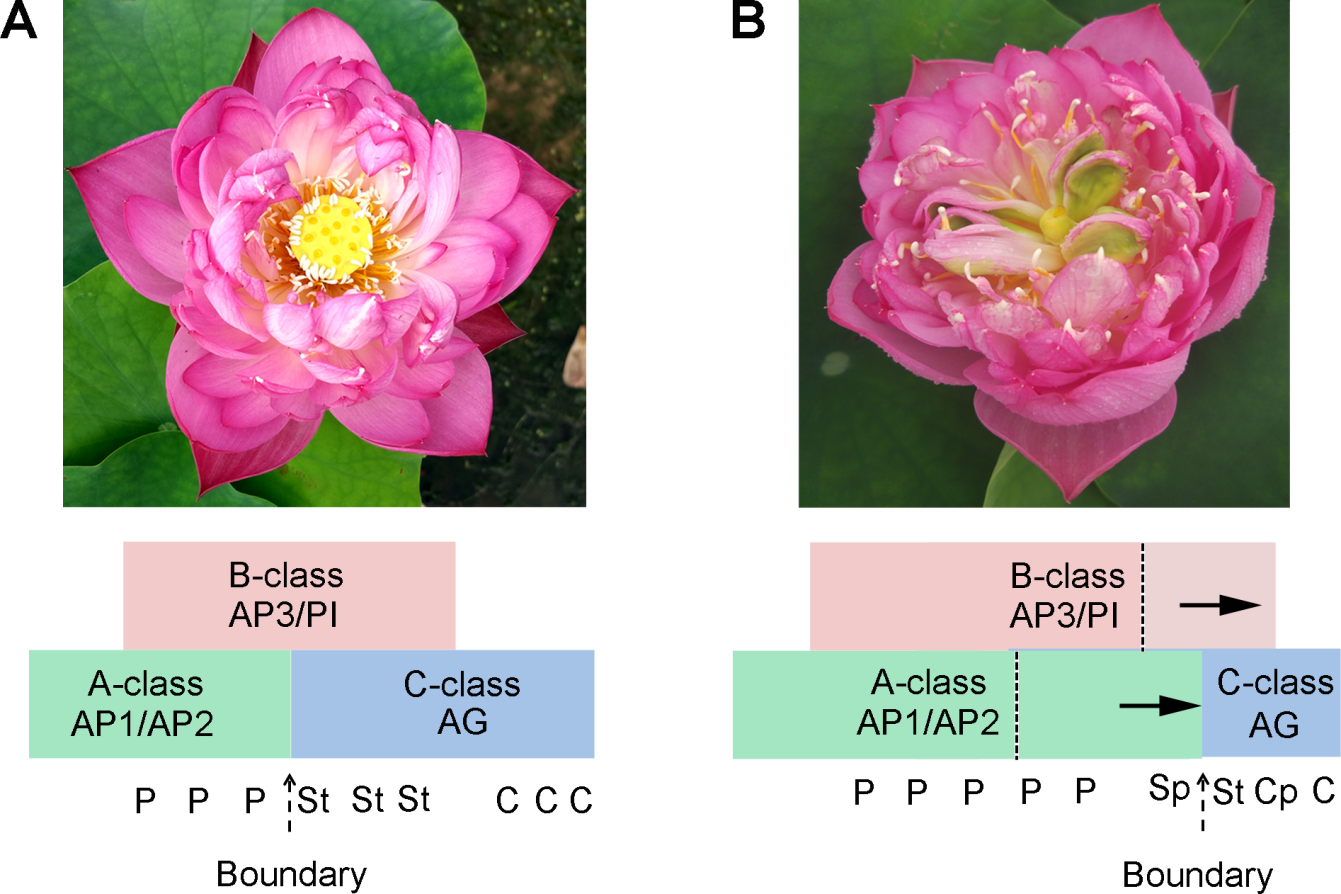
The proposed model in flower pattern of lotus. (A) Normal flower pattern in lotus. (B) Aberrant flower pattern in lotus. The petals/stamens/carpels boundaries are slide in the flowers.

Several candidate targets of MADS-box genes have known function in petal growth (Sablowski, 2015). In the floral classic ABC model, petal identity is specified by class A and B genes, stamen by class B and C genes, and carpel by class C genes. For Fig. S6, our results suggest that A-, B-, and C-class genes are involved in the petaloid formation, which is in agreement with previous studies. Deficiencies in inter-whorl boundaries can result in hybrid structures such as petal-stamens (Rebocho et al., 2017). The stamen petaloidy and carpel petaloidy in lotus may also be caused by defects in inter-whorl boundaries. In our previously study, the result show that there is antagonism between A-class genes and C-class genes (Lin et al., 2018). Following the past two-decade studies about floral organ identity genes, the gene model of lotus flower was summarized with a kind of supposition in Fig. 7. Our hypothesis proposed that not only a boundary between A- and C-class genes was shifted, but also the boundary of B- and C-class genes was changed in the double flower of louts. However, this hypothesis requires further verification. Further investigation on these boundary genes should be conducted to understand how they build the restricted expression pattern and perform functions in their complicated regulation network.

## Conclusion

This study was carried out to investigate the different transcriptomic dynamics resulting in abnormal flower morphology of lotus. Through comprehensive analysis, 1,568 DEGs related to carpel petaloidy were identified. Fifteen DEGs were validated by qRT-PCR. Several transcription factors and multiple genes involved in phytohormone pathways, especially related to the AUX signaling pathway, were found associated with carpel petaloidy formation in this study. Notably, the member of the MADS-box family, *AG* being a floral homeotic gene played a key role in floral organ petaloidy.

##  Supplemental Information

10.7717/peerj.12322/supp-1 Supplemental Information 1 Correlation of FPKMs of all DEGs in pair-wise comparison among C, Cp, and P

10.7717/peerj.12322/supp-2 Supplemental Information 2 GO term (A) and KEGG pathway (B) significantly enriched in 1568 DEGs

10.7717/peerj.12322/supp-3 Supplemental Information 3 The most enriched GO terms of three clustered annotated DEGs (A) The most enriched GO terms of cluster 1 with 719 annotated DEGs. (B) The most enriched GO terms of cluster 2 with 211 annotated DEGs. (C) The most enriched GO terms of cluster 3 with 638 annotated DEGs.

10.7717/peerj.12322/supp-4 Supplemental Information 4 KEGG classification of three clustered annotated DEGs (A) KEGG classification of cluster 1 with 719 annotated DEGs. (B) KEGG classification of cluster 2 with 211 annotated DEGs. (C) KEGG classification of cluster 3 with 638 annotated DEGs.

10.7717/peerj.12322/supp-5 Supplemental Information 5 The expression pattern of ABC model genes in lotus Values represent the means and standard errors (SEs) of three biological replicates. Petal: P; Carpel petaloidy: Cp; Carpel: C; Stamen petaloidy: Sp; Stamen: St. The FPKM values in Sp and St by RNA-seq were also analyzed (clean data of Sp and St by RNA-seq were deposited in NCBI with PRJNA524054). Data were analyzed with one-way ANOVA and Tukey’s multiple comparison test, *p* < 0.05.

10.7717/peerj.12322/supp-6 Supplemental Information 6 Correlation of expression changes observed by RNA-seq (*X*-axis) and qRT-PCR (*Y*-axis)

10.7717/peerj.12322/supp-7 Supplemental Information 7 Primers used in this study

10.7717/peerj.12322/supp-8 Supplemental Information 8 Summary of RNA sequencing and assembly

10.7717/peerj.12322/supp-9 Supplemental Information 9 All gene annotations

10.7717/peerj.12322/supp-10 Supplemental Information 10 1568 annotated common DEGs

10.7717/peerj.12322/supp-11 Supplemental Information 11 KEGG pathway enrichment analysis of 1568 DEGs

10.7717/peerj.12322/supp-12 Supplemental Information 12 Data of qRT-PCR

## Abbreviations

ABA: Abscisic acid
AG: AGAMOUS
AP: APETALA
AUX: auxin
BR: Brassinosteroid
C: Carpel
Cp: Carpel petaloidy
CTK: Cytokinine
DEGs: Differentially expressed genes
ETH: Ethylene
FDR: False discovery rate
FPKM: Fragments per kilo base of transcript per million base pairs sequenced
GA: Gibberellin
GO: Gene ontology
JA: Jasmonic acid
KEGG: Kyoto encyclopedia of genes and genomes
KOBAS: KEGG orthology-based annotation system
LFY: LEAFY
MADS: MCM1, AG, DEFA and SRF
P: Petal
PI: PISITTALA
qRT-PCR: Quantitative real-time PCR
RIN: RNA integrity number
SA: Salicylic acid
SEM: Scanning electron microscope
Sp: Stamen petaloidy
St: Stamen
TFs: Transcript factors
WUS: WUSCHEL

## Additional Information and Declarations

### Data Availability

The following information was supplied regarding data availability: The RNA-seq data generated in this study are available at NCBI: PRJNA524054. The qRT-PCR data are available in a Supplemental File.
