# Supplementary figures and images for: Comparative transcriptomic analysis provides insight into carpel petaloidy in lotus (Nelumbo nucifera)
Source: PeerJ. 2021 Oct 25;9:e12322. doi: 10.7717/peerj.12322 (PMC8552788; doi:10.7717/peerj.12322)

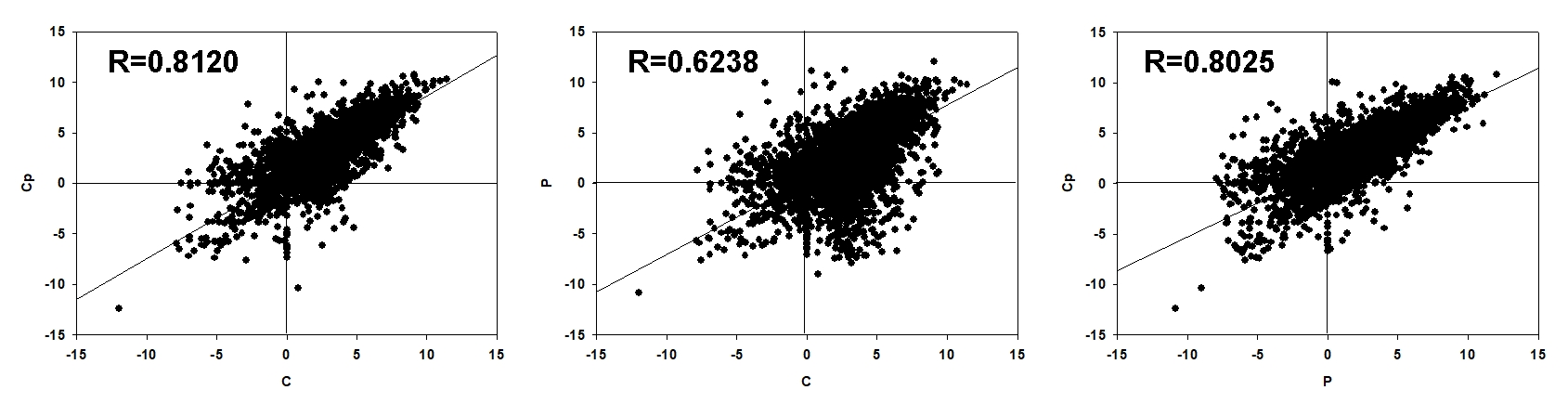

Supplement: Supplemental Information 1 [file peerj-09-12322-s001.jpg]

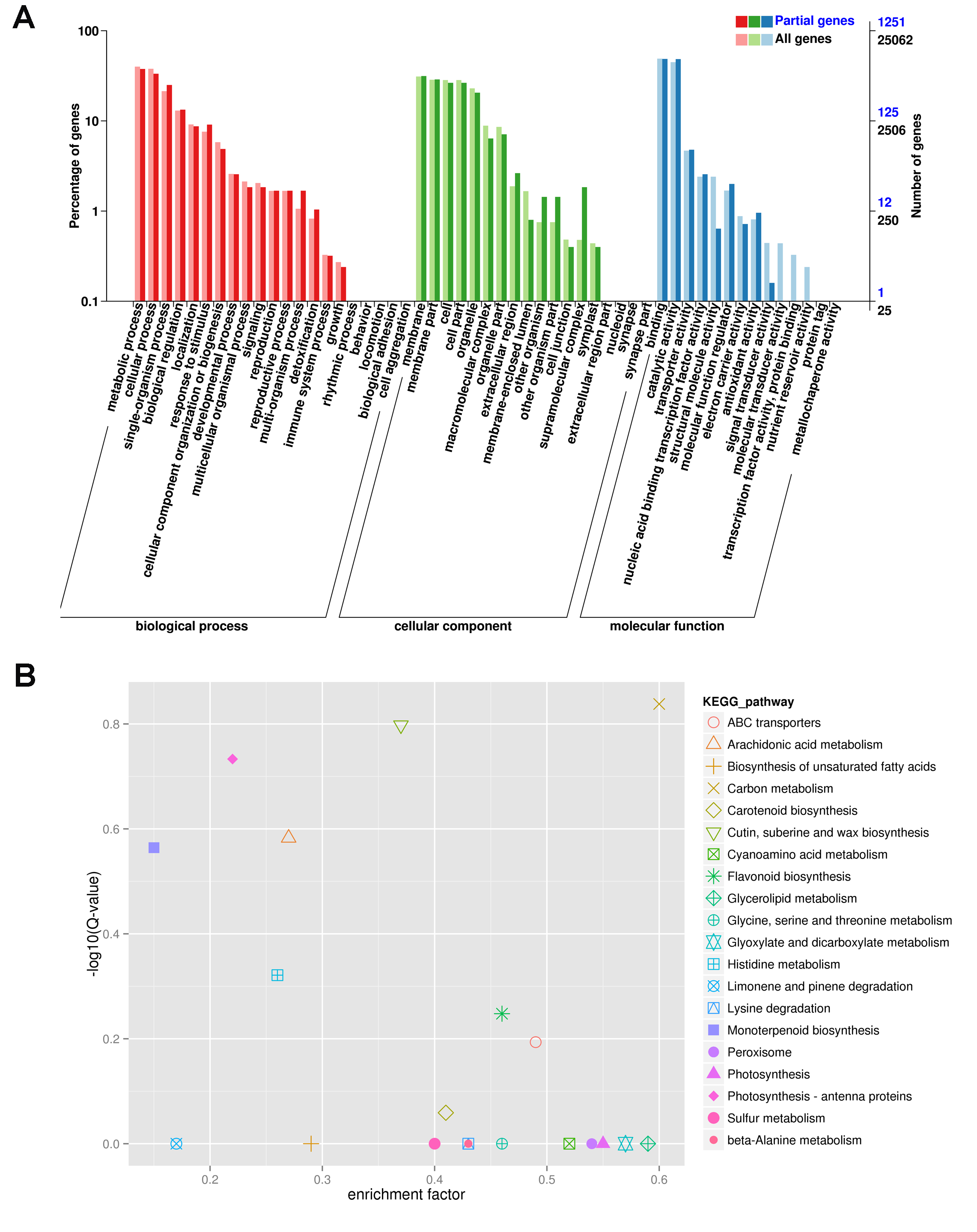

Supplement: Supplemental Information 2 [file peerj-09-12322-s002.png]

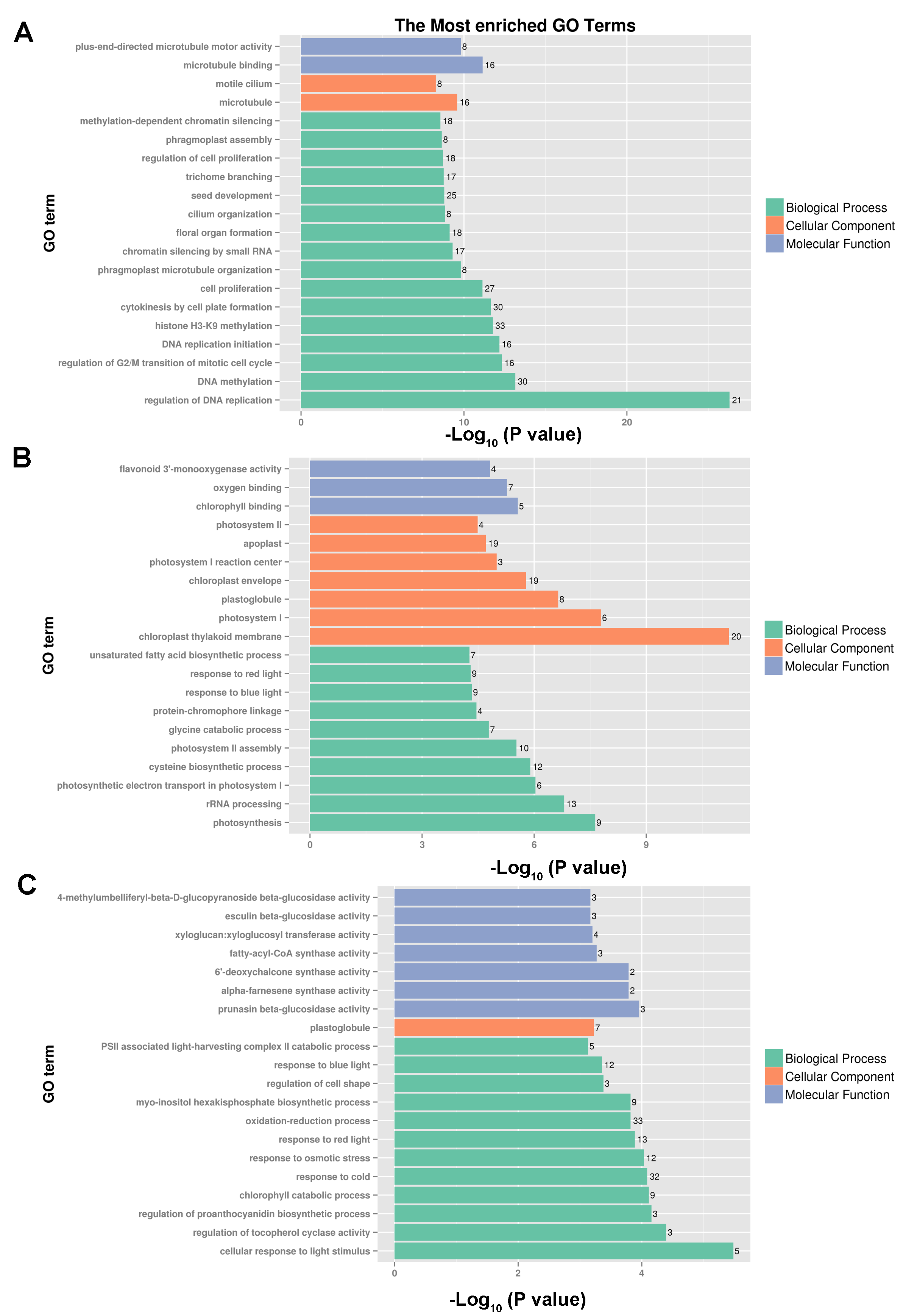

Supplement: Supplemental Information 3 — (A) The most enriched GO terms of cluster 1 with 719 annotated DEGs. (B) The most enriched GO terms of cluster 2 with 211 annotated DEGs. (C) The most enriched GO terms of cluster 3 with 638 annotated DEGs. [file peerj-09-12322-s003.png]

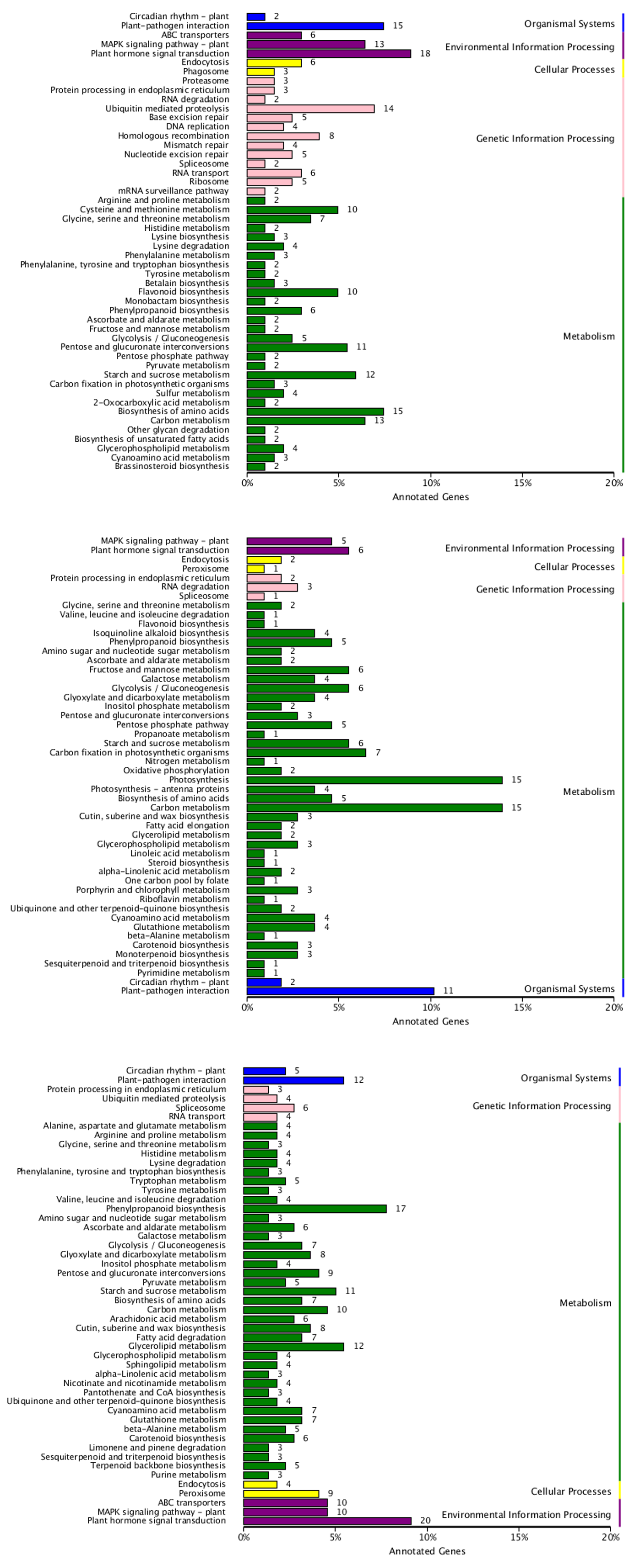

Supplement: Supplemental Information 4 — (A) KEGG classification of cluster 1 with 719 annotated DEGs. (B) KEGG classification of cluster 2 with 211 annotated DEGs. (C) KEGG classification of cluster 3 with 638 annotated DEGs. [file peerj-09-12322-s004.png]

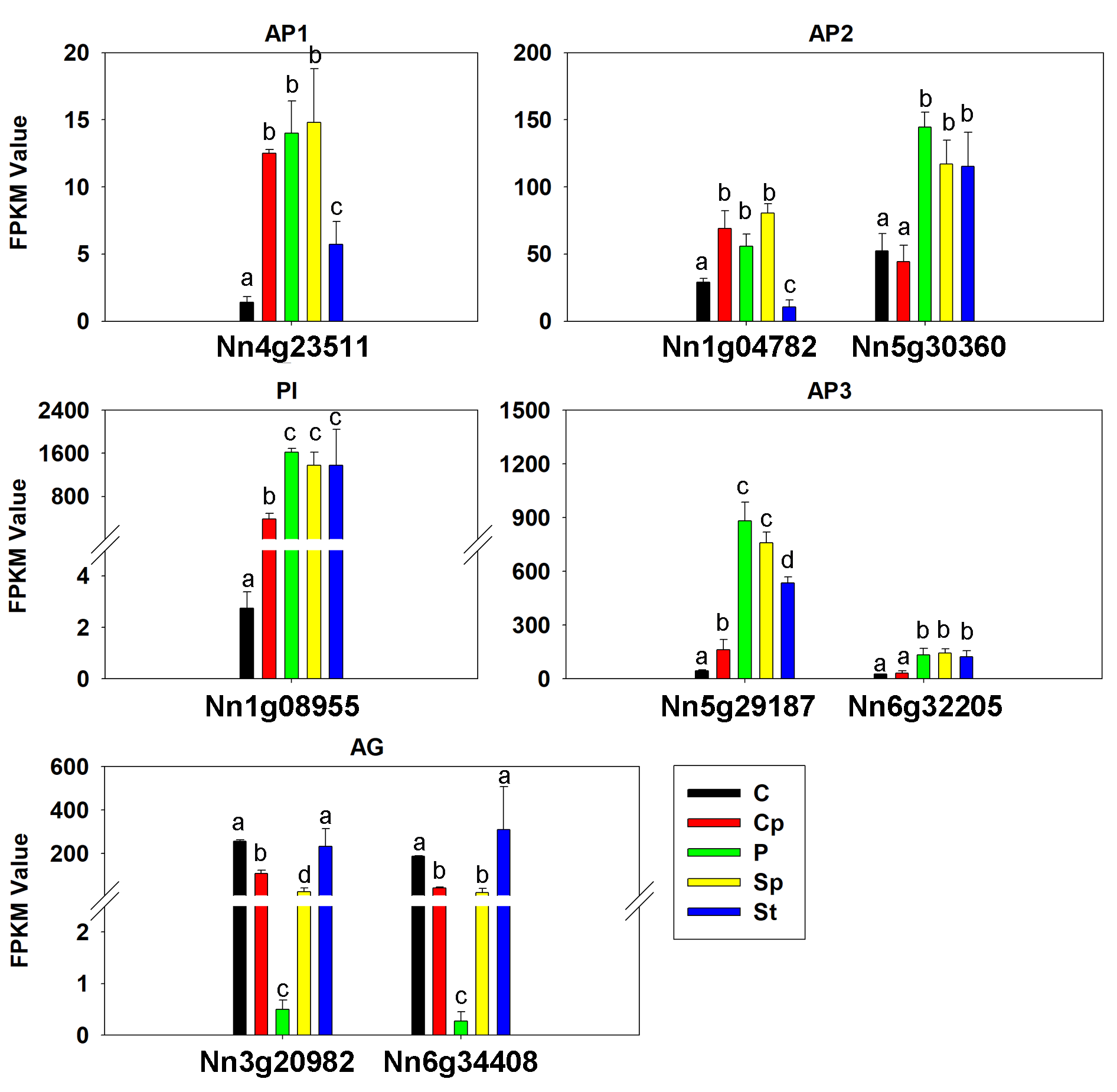

Supplement: Supplemental Information 5 — Values represent the means and standard errors (SEs) of three biological replicates. Petal: P; Carpel petaloidy: Cp; Carpel: C; Stamen petaloidy: Sp; Stamen: St. The FPKM values in Sp and St by RNA-seq were also analyzed (clean data of Sp and St by RNA-seq were deposited in NCBI with PRJNA524054). Data were analyzed with one-way ANOVA and Tukey’s multiple comparison test, p < 0.05. [file peerj-09-12322-s005.png]

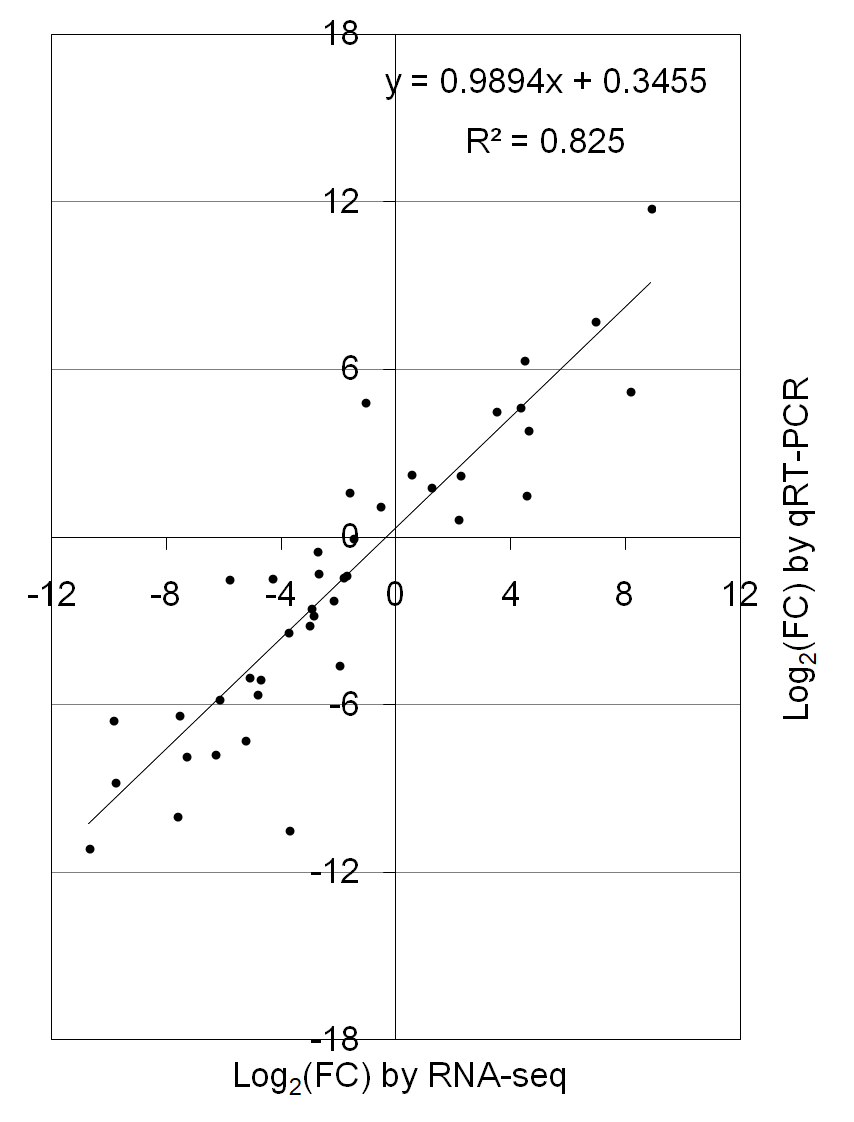

Supplement: Supplemental Information 6 [file peerj-09-12322-s006.png]
