# Supplementary material for: Comparative transcriptomic analysis provides insight into carpel petaloidy in lotus (Nelumbo nucifera)
Source: PeerJ. 2021 Oct 25;9:e12322. doi: 10.7717/peerj.12322 (PMC8552788; doi:10.7717/peerj.12322)
Supplement: Supplemental Information 7 [file peerj-09-12322-s007.docx]

**Table S1 Primers for qRT-PCR**

| Gene name | Forward primer （5’-3’） | Reverse primer （5’-3’） |
| --- | --- | --- |
| Nn4g23831 | AGAAAAACATCATTGAAACTCG | TGTCCTCCTACTCCTCCCA |
| Nn2g14034 | AAACGGTCCTCCCCTTCA | GGTCCCGCCATTCAGTAAC |
| Nn3g21830 | ACACCCATCACCCAGCAACT | TCTACTTCATCCGCCTCTCG |
| Nn3g20982 | TGGAGGGTAGATTGGAGAAAGG | TGGCGAGAGTAATGGTGGTTG |
| Nn2g10308 | CTTCTCTATGCGTTTTGTGTATC | GTCTTGAGGTTCTTTCTTGGTG |
| Nn2g10859 | GGCTGTGAAACTATTGAACCTG | TACCACTGAACTTCTCCTCGTC |
| Nn2g13182 | TCTCTTAGTTGCGATGACCG | TCCTTCCACCCATAGTAGCC |
| Nn3g18926 | TAAGATGTGGAAAGAGTTGC | AGGTAGTCTCCCTGCGATGA |
| Nn2g13807 | CAGGCAGGACTGACAATGATA | TGGAGATGAGTTGGTAAGGCT |
| Nn2g11466 | CGACTCCATTCCAGCCCTAC | CTCCACCAACTCAACCTTTCC |
| Nn2g11602 | GATTACCAAGGCAACCATTTC | GTGTGGACAAGAGCAGCAAGT |
| Nn4g22107 | ACACTATGTGCTGTTCTGGGA | CCGATGGACTTGTCTTTGC |
| Nn2g15879 | GAAGATGGTGGGGAAGGAC | TCTGTGATAGACAATGGGTGC |
| Nn5g30798 | GGCAACGCCAGTTTCTATC | CGTCGCACTTGTCACCTATG |
| Nn3g18142 | AAAGCCAGGTGTGCCGAG | TGCCCTTCAGATACGATAGAGC |
