# Supplementary material for: Comparative transcriptomic analysis provides insight into carpel petaloidy in lotus (Nelumbo nucifera)
Source: PeerJ. 2021 Oct 25;9:e12322. doi: 10.7717/peerj.12322 (PMC8552788; doi:10.7717/peerj.12322)
Supplement: Supplemental Information 8 [file peerj-09-12322-s008.docx]

**Table S2 Summary of RNA sequencing and assembly**

| **Samples** | **Clean Reads** | **Map Rate** | **GC Content** | **≥Q30%** |
| --- | --- | --- | --- | --- |
| C1 | 21,328,777 | 94.36% | 45.49% | 92.09% |
| C2 | 20,756,335 | 93.92% | 45.63% | 92.38% |
| C3 | 24,382,509 | 94.41% | 45.52% | 92.72% |
| Cp1 | 20,234,220 | 94.13% | 45.60% | 92.61% |
| Cp2 | 24,501,034 | 93.21% | 45.64% | 92.36% |
| Cp3 | 23,168,691 | 934.11% | 45.59% | 92.66% |
| P1 | 22,605,631 | 94.22% | 45.48% | 92.41% |
| P2 | 20,647,526 | 93.68% | 45.36% | 92.23% |
| P3 | 21,922,069 | 93.98% | 44.89% | 92.61% |
