# Supplementary material for: Comparative transcriptomic analysis provides insight into carpel petaloidy in lotus (Nelumbo nucifera)
Source: PeerJ. 2021 Oct 25;9:e12322. doi: 10.7717/peerj.12322 (PMC8552788; doi:10.7717/peerj.12322)
Supplement: Supplemental Information 11 [file peerj-09-12322-s011.docx]

**Table S5 KEGG pathway enrichment analysis of 1568 DEGs**

| **Pathway** | **Pathway ID** | **Genes with pathway annotation** |
| --- | --- | --- |
| Glycosylphosphatidylinositol (GPI)-anchor biosynthesis | ko00563 | 3 |
| Non-homologous end-joining | ko03450 | 1 |
| Starch and sucrose metabolism | ko00500 | 29 |
| Photosynthesis | ko00195 | 16 |
| Riboflavin metabolism | ko00740 | 3 |
| Butanoate metabolism | ko00650 | 2 |
| 2-Oxocarboxylic acid metabolism | ko01210 | 3 |
| Circadian rhythm - plant | ko04712 | 9 |
| Ubiquinone and other terpenoid-quinone biosynthesis | ko00130 | 6 |
| Carbon fixation in photosynthetic organisms | ko00710 | 11 |
| mRNA surveillance pathway | ko03015 | 3 |
| Homologous recombination | ko03440 | 9 |
| Ether lipid metabolism | ko00565 | 3 |
| Autophagy - other | ko04136 | 1 |
| Brassinosteroid biosynthesis | ko00905 | 4 |
| Proteasome | ko03050 | 3 |
| Anthocyanin biosynthesis | ko00942 | 1 |
| Aminoacyl-tRNA biosynthesis | ko00970 | 2 |
| Lysine degradation | ko00310 | 8 |
| Glycosphingolipid biosynthesis - ganglio series | ko00604 | 1 |
| Nicotinate and nicotinamide metabolism | ko00760 | 4 |
| Sphingolipid metabolism | ko00600 | 4 |
| Glycine, serine and threonine metabolism | ko00260 | 12 |
| Ribosome biogenesis in eukaryotes | ko03008 | 1 |
| Flavonoid biosynthesis | ko00941 | 14 |
| Betalain biosynthesis | ko00965 | 4 |
| Base excision repair | ko03410 | 5 |
| Other glycan degradation | ko00511 | 4 |
| Taurine and hypotaurine metabolism | ko00430 | 1 |
| Purine metabolism | ko00230 | 5 |
| Folate biosynthesis | ko00790 | 2 |
| Mismatch repair | ko03430 | 4 |
| N-Glycan biosynthesis | ko00510 | 2 |
| Glyoxylate and dicarboxylate metabolism | ko00630 | 14 |
| ABC transporters | ko02010 | 16 |
| Glycolysis / Gluconeogenesis | ko00010 | 18 |
| Thiamine metabolism | ko00730 | 1 |
| Various types of N-glycan biosynthesis | ko00513 | 2 |
| Monobactam biosynthesis | ko00261 | 2 |
| Sesquiterpenoid and triterpenoid biosynthesis | ko00909 | 4 |
| Monoterpenoid biosynthesis | ko00902 | 4 |
| Carbon metabolism | ko01200 | 38 |
| Pyruvate metabolism | ko00620 | 7 |
| Phenylalanine metabolism | ko00360 | 5 |
| Oxidative phosphorylation | ko00190 | 2 |
| Carotenoid biosynthesis | ko00906 | 10 |
| Peroxisome | ko04146 | 12 |
| Cysteine and methionine metabolism | ko00270 | 13 |
| Alanine, aspartate and glutamate metabolism | ko00250 | 4 |
| Arginine biosynthesis | ko00220 | 1 |
| Flavone and flavonol biosynthesis | ko00944 | 3 |
| MAPK signaling pathway - plant | ko04016 | 28 |
| Arginine and proline metabolism | ko00330 | 7 |
| Ubiquitin mediated proteolysis | ko04120 | 18 |
| Pentose and glucuronate interconversions | ko00040 | 23 |
| Phagosome | ko04145 | 4 |
| DNA replication | ko03030 | 4 |
| Tropane, piperidine and pyridine alkaloid biosynthesis | ko00960 | 3 |
| Inositol phosphate metabolism | ko00562 | 8 |
| Pantothenate and CoA biosynthesis | ko00770 | 4 |
| Ribosome | ko03010 | 6 |
| Glycosphingolipid biosynthesis - lacto and neolacto series | ko00601 | 1 |
| Terpenoid backbone biosynthesis | ko00900 | 5 |
| Arachidonic acid metabolism | ko00590 | 7 |
| Stilbenoid, diarylheptanoid and gingerol biosynthesis | ko00945 | 3 |
| Fatty acid elongation | ko00062 | 5 |
| Glycosphingolipid biosynthesis - globo and isoglobo series | ko00603 | 2 |
| Valine, leucine and isoleucine biosynthesis | ko00290 | 1 |
| Photosynthesis - antenna proteins | ko00196 | 6 |
| Glycerolipid metabolism | ko00561 | 15 |
| alpha-Linolenic acid metabolism | ko00592 | 6 |
| Protein processing in endoplasmic reticulum | ko04141 | 8 |
| Amino sugar and nucleotide sugar metabolism | ko00520 | 7 |
| Cutin, suberine and wax biosynthesis | ko00073 | 12 |
| One carbon pool by folate | ko00670 | 3 |
| Fatty acid biosynthesis | ko00061 | 1 |
| Nitrogen metabolism | ko00910 | 5 |
| Glycosaminoglycan degradation | ko00531 | 1 |
| Histidine metabolism | ko00340 | 6 |
| Zeatin biosynthesis | ko00908 | 2 |
| Sulfur metabolism | ko00920 | 6 |
| Cyanoamino acid metabolism | ko00460 | 14 |
| Pyrimidine metabolism | ko00240 | 4 |
| Diterpenoid biosynthesis | ko00904 | 2 |
| Biosynthesis of amino acids | ko01230 | 27 |
| Pentose phosphate pathway | ko00030 | 8 |
| Fatty acid metabolism | ko01212 | 4 |
| Biosynthesis of unsaturated fatty acids | ko01040 | 4 |
| Galactose metabolism | ko00052 | 9 |
| Limonene and pinene degradation | ko00903 | 3 |
| Basal transcription factors | ko03022 | 1 |
| Tyrosine metabolism | ko00350 | 6 |
| Endocytosis | ko04144 | 12 |
| Isoflavonoid biosynthesis | ko00943 | 1 |
| C5-Branched dibasic acid metabolism | ko00660 | 1 |
| Valine, leucine and isoleucine degradation | ko00280 | 6 |
| Linoleic acid metabolism | ko00591 | 2 |
| Fructose and mannose metabolism | ko00051 | 9 |
| Porphyrin and chlorophyll metabolism | ko00860 | 6 |
| Fatty acid degradation | ko00071 | 7 |
| Phosphatidylinositol signaling system | ko04070 | 3 |
| Isoquinoline alkaloid biosynthesis | ko00950 | 8 |
| Phenylpropanoid biosynthesis | ko00940 | 28 |
| RNA transport | ko03013 | 10 |
| Phenylalanine, tyrosine and tryptophan biosynthesis | ko00400 | 5 |
| Glutathione metabolism | ko00480 | 12 |
| Glucosinolate biosynthesis | ko00966 | 1 |
| Lysine biosynthesis | ko00300 | 3 |
| Ascorbate and aldarate metabolism | ko00053 | 10 |
| Spliceosome | ko03040 | 9 |
| Propanoate metabolism | ko00640 | 4 |
| beta-Alanine metabolism | ko00410 | 7 |
| RNA degradation | ko03018 | 7 |
| Tryptophan metabolism | ko00380 | 6 |
| Glycerophospholipid metabolism | ko00564 | 11 |
| Nucleotide excision repair | ko03420 | 5 |
| Plant hormone signal transduction | ko04075 | 44 |
| Steroid biosynthesis | ko00100 | 2 |
| Plant-pathogen interaction | ko04626 | 38 |
